# Fear of Becoming Infected and Fear of Doing the Wrong Thing – Cross-Cultural Adaptation and Further Validation of the Multidimensional Assessment of COVID-19-Related Fears (MAC-RF)

**DOI:** 10.32872/cpe.6137

**Published:** 2022-03-31

**Authors:** Branka Bagarić, Nataša Jokić-Begić

**Affiliations:** 1 Croatian Association for Behavioral-Cognitive Therapies (CABCT), Zagreb, Croatia; 2 Department of Psychology, Faculty of Humanities and Social Sciences, University of Zagreb, Zagreb, Croatia; Philipps-University of Marburg, Marburg, Germany

**Keywords:** COVID-19 fear, MAC-RF, health anxiety, cyberchondria, scale validity

## Abstract

**Background:**

With the COVID-19 infection speeding around the world, many experience fear and anxiety. To detect those at risk of psychopathology and provide treatment, valid instruments are needed. The aim of this study was to cross-culturally validate the theory-based instrument Multidimensional Assessment of COVID-19-Related Fears (MAC-RF) in Croatian and to further examine the scale’s validity by exploring its relationship with relevant constructs.

**Method:**

A total of 477 participants completed an online survey during a rapid rise in new daily COVID-19 cases in Croatia and while new restrictions were being imposed.

**Results:**

MAC-RF had a stronger association with health anxiety, cyberchondria, and anxiety sensitivity compared to depression, attesting to its convergent and divergent validity. However, a 2-factor structure was revealed in this sample: Fear of infection and Fear of using an inadequate strategy in dealing with pandemic. Fear of infection had a stronger association with health anxiety and COVID-19 anxiety and was a better predictor of COVID-19 related protective health behaviors. Fear of choosing an inadequate strategy had a stronger association with cyberchondria, fear of consequences of the epidemic on mental health, as well as financial consequences, and loss of civil liberties.

**Conclusion:**

Fear of infection captures negative emotional states due to feared consequences on personal somatic health and the health of loved ones, while Fear of choosing an inadequate strategy in dealing with the pandemic reflects a metacognitive aspect. Treatments may have to target both aspects of COVID-19 related fear.

## Highlights

- MAC-RF is a theory-based instrument for measuring COVID-19 related fears.
- MAC-RF has two factors: Fear of infection and Fear of using an inadequate strategy in dealing with the pandemic.
- First factor had a stronger association with heath anxiety and protective health behaviors.
- Second factor had a stronger association with cyberchondria and fear for mental health.

With over 200 million people infected and over 4 million dead from COVID-19 around the world, in addition to the social restrictions that affect our everyday life, the rise of fear, anxiety and distress is to be expected. Although somatic health has the focus of attention, it has become evident that psychological consequences of the epidemic may be equally severe (e.g., Kumar & Nayar, 2021), but more difficult to detect. The development of instruments that measure psychopathology associated with COVID-19 is an important step in identifying individuals at risk and developing treatments.

During 2020, several measures focused on different aspects of negative psychological reactions to COVID-19 pandemic emerged. The *Fear of COVID-19 Scale* (FCV-19S; Ahorsu et al., 2020) is a 7-item instrument measuring a single factor. The *Coronavirus Anxiety Scale* (CAS; Lee, 2020) and *COVID-19 Anxiety Scale* (CAS5; Lauri-Korajlija & Jokić-Begić, 2020) are both 5-item scales, both measuring a single factor. The *COVID-19 Anxiety Syndrome Scale* (C-19ASS; Nikčević & Spada, 2020) is a 9-item scale measuring two factors: Perseveration and Avoidance. The *COVID-19 Phobia Scale* (C19P-S; Arpaci et al., 2020) is a 20-item instrument measuring four factors: Psychological, Psycho-somatic, Economic, and Social. Finally, the *COVID Stress Scales* (CSS; Taylor et al., 2020) is a 36-item instrument, measuring 5-factors: Danger and contamination fears, Fears about economic consequences, Xenophobia, Compulsive checking and reassurance seeking, and Traumatic stress symptoms about COVID-19. Considering differences in the breadth of focus of these scales, it is not surprising that different structures of the underlying construct have been reported.

Determining which aspects of psychological experience should be captured in such an instrument might be aided by a theoretical framework. This type of theory-based instrument has been recently developed – the *Multidimensional Assessment of COVID-19-Related Fears* (MAC-RF). According to the model behind the MAC-RF, as proposed by Schimmenti, Billieux, and Starcevic (2020), four mutually linked domains are involved in fear experiences during a pandemic: bodily, relational, cognitive, and behavioral. These domains are assumed to be organized in a dialectical structure. As such, the bodily domain involves 1) fear of the body and 2) fear for the body; the interpersonal domain involves 3) fear of others and 4) fear for others; the cognitive domain involves 5) fear of knowing and 6) fear of not knowing; and the behavioral domain involves 7) fear of action and 8) fear of inaction. The MAC-RF appears to be a useful instrument in assessing pathological levels of fear during pandemics (Schimmenti, Starcevic, et al., 2020). However, more studies of its validity are needed.

Fear related to COVID-19 is found to be associated with general psychopathology, general anxiety, health anxiety and depression (Ahorsu et al., 2020; Schimmenti, Starcevic, et al., 2020; Taylor et al., 2020), functional impairment and dysfunctional coping (Lee, 2020; Nikčević & Spada, 2020). Several studies have suggested that anxiety sensitivity (fear of consequences of anxiety) and cyberchondria (excessive online search for health information followed by distress) might explain problematic responses to pandemic (Hashemi et al., 2020; Manning et al., 2021; McKay et al., 2020). Specifically, it is suggested that because people with high anxiety sensitivity believe their physical sensations produced by anxiety to be harmful, they might experience more distress. Those who are more distressed may be prone to searching for information about their health on the internet, resulting in even more distress due to the frightening information they encounter (Hashemi et al., 2020). Anxiety sensitivity may be associated with Fear of and for the body, whereas cyberchondria may reflect the Fear of knowing and not knowing, as proposed in Schimmenti, Billieux, and Starcevic’s (2020) model.

Although the pandemic is a global crisis, there are differences in how a given country will respond to an outbreak in the type and duration of restrictions, in addition to economic, societal and cultural differences, which may affect how individuals experience and cope with pandemic. Hence, the aim of this study was to: 1) cross-culturally validate the Croatian version of the MAC-RF; and 2) to further examine the scale validity by exploring its relationship with relevant constructs: health anxiety, anxiety sensitivity, cyberchondria, COVID-19 safety behaviors, health care use and fear of different COVID consequences.

We predicted that the MAC-RF would: 1) have a single-factor solution, as reported by the scale’s authors; 2) be associated with general psychopathology, as reported by the authors; 3) have a strong correlation with a previously validated measure of fear of COVID-19 (concurrent validity); 4) have a stronger association with health anxiety, anxiety sensitivity and cyberchondria as compared to depression (converged and divergent validity); and 5) have a positive association with COVID-19 safety behaviors, health care utilization and fear of different COVID consequences.

The results of this study would inform the possibility of cross-cultural generalization of findings in the field. Furthermore, this study may shed further light on possible predictors, mechanisms and consequences of fear of pandemic, and hence inform future experimental, longitudinal and intervention research.

## Method

### Participants

There were 477 participants in this study (an additional 25 participants filled out sociodemographic items only and were excluded from the data set), of which 74.8% were female. The mean age was 34.70 years (*SD* = 9.71; *Total range [TR]* = 18-71). With respect to education, 25.99% were high school graduates, 10.27% held a bachelor’s degree, 53.46% held a master’s degree, and 10.27% held a PhD. Furthermore, 8.17% of participants were employed in the health care system and an additional 1.26% were trained in health sciences but were not employed in the health system. A total of 11.94% of participants reported suffering from a chronic condition, most commonly from thyroid diseases, asthma, allergies, depression, diabetes and anxiety. In regards to their experiences with COVID-19, most participants reported personally knowing five (mode = 5; *M* = 7.05; *TR* = 0-200) people who tested positive for COVID-19, 12.88% reported they themselves had tested positive for COVID-19 at some point, and an additional 18.03% believed they had had COVID-19 although this was not confirmed by a test. Participants who tested positive for COVID-19 on average estimated their symptoms to be mild (*M* = 32.04, *SD* = 22.90, *TR* = 0-83) and this experience to be only mildly uncomfortable (*M* = 34.63, *SD* = 27.88, *TR* = 0-100), although there was great variability in responses.

### Instruments

#### Multidimensional Assessment of COVID-19-Related Fears (MAC-RF)

The MAC-RF (Schimmenti, Starcevic, et al., 2020) is a newly developed 8-item measure of clinically relevant domains of fear during the COVID-19 pandemic. Items cover four domains of fear: bodily, relational, cognitive, and behavioral and are scored on a scale ranging from 0 (very unlike me) to 4 (very like me). Authors reported a single-factor structure, satisfactory reliability (Cronbach’s alpha = .84), whereas convergent validity was based on its positive correlation with overall psychopathology. Cronbach’s α in this study was .72.

#### COVID-19 Anxiety Scale (CAS5)

The CAS5 (Lauri-Korajlija & Jokić-Begić, 2020) is a recently developed 5-item instrument inspired by the *Swine Flu Anxiety Scale* (Wheaton et al., 2012) that assesses worrying about COVID, perceived likelihood of contracting the virus (oneself and others), perceived severity of infection, and the degree to which a person believes COVID is a more serious illness than the flu. Each item is rated on a 5-point scale (1 = not at all; 5 = very much). Authors reported a Cronbach’s alpha coefficient of 0.76 and 0.78. This is the only COVID-19 distress scale that has been validated in the Croatian language. Cronbach’s α in this study was 0.74.

#### DSM-5 Self-Rated Level 1 Cross-Cutting Symptom Measure—Adult (CCSM)

The CCSM (APA, 2013) consists of 23 questions assessing 13 psychiatric domains: depression, anger, mania, anxiety, somatic symptoms, suicidal ideation, psychosis, sleep problems, memory problems, repetitive thoughts and behaviors, dissociation, personality functioning, and substance use. The respondent rates their experiences during the last two weeks on a scale ranging from 0 (none or not at all) to 4 (severe or nearly every day). The instrument has demonstrated good psychometric properties (Narrow et al., 2013). Cronbach’s α in this study was 0.89.

#### Short Health Anxiety Inventory (SHAI)

The SHAI (Salkovskis et al., 2002) consists of 18 items measuring two factors: health anxiety (14 items) and fear of negative consequences of illness (4 items). It uses a multiple choice format with response options ranging from 0 to 3 (from no pathology to severe pathology; Alberts et al., 2013). The instrument demonstrated good psychometric properties in both clinical and non-clinical samples (Alberts et al., 2013). Cronbach’s α in this study was 0.85 for the health anxiety factor and 0.86 for the full scale.

#### Short Cyberchondria Scale (SCS)

The SCS (Jokić-Begić et al., 2019) consists of four items (e.g., After searching for health information, I feel frightened) rated on a 5-point Likert scale. The SCS has demonstrated satisfactory psychometric properties, has a unidimensional structure and measures the same latent construct as the significantly longer instrument developed by McElroy and Shevlin (2014); *Cyberchondria Severity Scale*, (Jokić-Begić et al., 2019). Cronbach’s α in this study was 0.80.

#### Anxiety Sensitivity Index – 3 (ASI-3)

The ASI-3 (Taylor et al., 2007) consists of 18 items measuring fear of anxiety and its consequences that are rated from 0 (very little) to 4 (very much). The ASI has three subscales measuring the fear of physical (It scares me when I become short of breath), cognitive (When I feel “spacey” or spaced out I worry that I may be mentally ill) and social (When I tremble in the presence of others, I fear what people might think of me) aspects of anxiety. ASI has demonstrated good psychometric properties. Cronbach’s α in this study was 0.92.

#### Depression, Anxiety and Stress Scale 21 (DASS)

The DASS (Lovibond & Lovibond, 1993) is a short form of the original DASS instrument and consist of 21 items measuring depression, anxiety, and stress during the last week, each rated on a scale ranging from 0 (Did not apply to me at all) to 3 (Applied to me very much or most of the time). Only the Depression subscale was used in this study (DASS-D), which consists of seven items describing dysphoria, hopelessness, lack of interest etc. (e.g., I couldn’t seem to experience any positive feeling at all). All three subscales demonstrated good psychometric properties in both clinical and non-clinical populations (Parkitny & McAuley, 2010). Cronbach’s α in this study was 0.92.

#### COVID Safety Behavior Checklist (CSBC)

The CSBC (Lauri-Korajlija & Jokić-Begić, 2020) consists of 13 items measuring safety behaviors that people engage in to avoid COVID infection, such as thorough and frequent hand washing, avoiding people that appear ill, avoiding leaving home etc., each rated on a 5-point scale (1 = not at all; 5-very much). The CSB was inspired by the *Ebola Safety Behavior Checklist* (Blakey et al., 2015). Cronbach’s α in this study was 0.86.

#### Health Care Use (HCU)

HCU was measured using a single item where participants assess the number of doctor visits (both GP and specialists) they attended in the last two months.

#### Fear of COVID-19 Consequences (FCCC)

FCCC was developed for the purposes of this study and consisted of six items covering fear of consequences on: physical health, mental health, loved ones’ health, financial loss, loss of civil liberties and disturbed relationships. Respondents rated how much they feared each of these consequences from 1 (very little) to 5 (very much).

### Procedure

We followed the procedure for instrument cross-validation described in the literature (van Widenfelt et al., 2005). The MAC-RF was first translated into Croatian by the two authors (Professor in clinical psychology and a doctoral student in clinical psychology) and by another colleague (Professor in biological psychology). All three versions were reviewed and compared and a final version was agreed upon. Next, a bilingual Professor in Health Psychology translated the final version back into English. Small differences were discussed by all four psychologists and minor alterations were made. This revised version was assessed by another two colleagues: a psychotherapist with a PhD in clinical psychology and a doctoral student in cognitive psychology. Neither reviewer found any issues. Finally, this version was completed and its content discussed by a small sample of laypersons known to the researchers, who found the items clear and easy to respond to.

Data were collected via an online survey using the SurveyMonkey software. This survey consisted of the aforementioned instruments and several questions regarding sociodemographic data and experiences with COVID-19 described in the “participants” section. The data collection period was limited to four weeks from the date the survey was published. Data was collected during the second wave of the COVID-19 pandemic in Croatia (November and December 2020), when a steady rise in new daily cases was being registered and new restrictions were being introduced. The survey was advertised using social media (several open groups dealing with different topics), the website of a CBT counseling center in Croatia and the authors’ personal contacts.

This study was approved by the Ethical committee of Department of Psychology, Faculty of Humanities and Social Sciences, University of Zagreb (EPOP – 2021 – 005).

### Data Analyses

Analyses were performed using the Lavaan R package (Rosseel et al., 2017). To explore the underlying structure of the MAC-RF, we performed confirmatory factor analyses. To determine the fit of the model, several goodness-of-fit criteria were used: the standard root mean square residual (SRMR), the root mean square error of approximation (RMSEA) with 90% confidence intervals, and the comparative fit index (CFI). A model is considered to have a good fit to the data if the SRMR is close to or below 0.08, if RMSEA is close to or below 0.06, (the upper limit of the 90% RMSEA confidence interval should be below 0.10), and if CFI is close to or above 0.95 (Brown, 2015; Hu & Bentler, 1999; Kline, 2015). To explore scale’s reliability and validity, we calculated Cronbach’s alpha and correlations with relevant measures.

## Results

Descriptive data for the MAC-RF items is presented in Table 1.

**Table 1:** Descriptive Statistics for MAC-RF Items

Item and domain	*M (SD)*	*TR*	Skewness	Kurtosis
1. Fear of the body	1.17 (1.13)	0-4	.643	-.612
2. Fear for the body	1.60 (1.29)	0-4	.133	-1.326
3. Fear of others	1.93 (1.29)	0-4	-.156	-1.271
4. Fear for others	2.55 (1.24)	0-4	-.754	-.464
5. Fear of knowing	1.82 (1.34)	0-4	-.009	-1.264
6. Fear of not knowing	0.55 (0.86)	0-3	1.436	1.000
7. Fear of action	0.80 (1.05)	0-4	1.110	.122
8. Fear of inaction	1.05 (1.17)	0-4	.724	-.788
Total score	11.48 (5.47)	0-27	.11	-.56

### Preliminary Analysis

Because it was treated as a single question by the program, there were no missing data within the MAC-RF matrix. Single multivariate and nine univariate outliers were detected and subsequently omitted from the data set. No indications of collinearity were detected (maximum variance inflation factor value = 2.11; minimum tolerance value = .47). All items were non-normally distributed (Kolmogorov-Smirnov *Z* = 4.16-8.52, all *p* values < .001).

### Model Generation

We specified three alternative models: a single factor model suggested by the authors, a 4-factor model with each domain of fear - bodily, relational, cognitive, and behavioral - comprising its own factor, and a 2-factor model with fear of infection comprising one factor (Items 1-4; fear for/of the body, fear of/for others) and fear of choosing an inadequate strategy in dealing with pandemic comprising the other factor (Items 5-8; fear of knowing and not knowing, fear of action/inaction).

### Confirmatory Factor Analyses

Due to non-normal data, the maximum likelihood estimation method with robust standard errors (MLR) was employed (Brown, 2015). MLR is recommended for variables that have five or more categories (Rhemtulla, Brosseau-Liard, & Savalei, 2012).

According to the proposed criteria (Brown, 2015; Hu & Bentler, 1999), the goodness‐of‐fit indices for three tested models suggested that a single factor solution fits the data poorly, whereas 2- and 4-factor solutions both provided a good fit to the data (Table 2). Since the difference in the fit of these two models was not statistically significant, χ^2^(5) = 4.08, *p* = 0.54, we preserved the more parsimonious 2-factor model.

**Table 2:** Goodness of Fit Indices for the Three Tested CFA Models of MAC-RF (N = 477)

Model	χ^2^ (*df*)	*p* (χ^2^)	SRMR	RMSEA [90% CI]	CFI
1-factor	204.13 (20)	< .001	0.10	0.14 [0.12, 0.16]	0.78
2-factor	32.06 (19)	.031	0.04	0.04 [0.01, 0.06]	0.99
4-factor	28.27 (14)	.013	0.03	0.05 [0.02, 0.07]	0.98

*Note.* SRMR = Standardized Root Mean Square Residual; RMSEA = The Root Mean Square Error of Approximation; CFI = comparative fit index.

**Figure 1:**
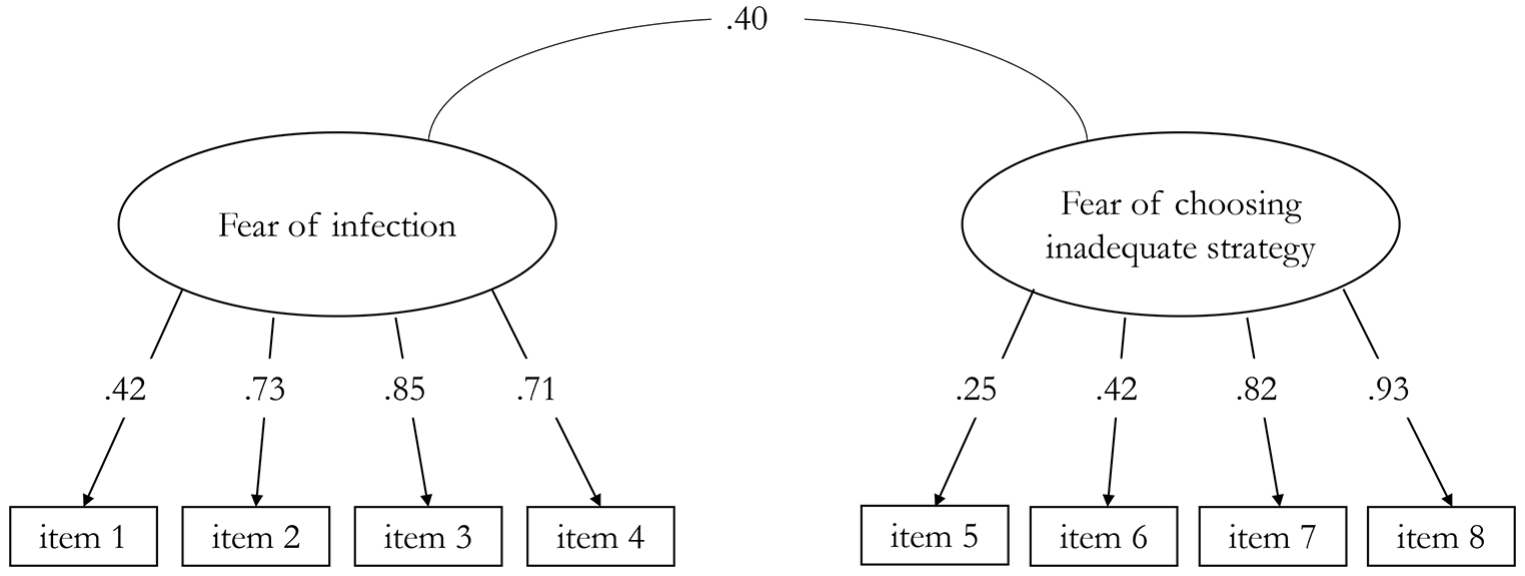
Standardized Parameter Estimates of the Accepted 2-Factor Model of the MAC-RF (N = 477) *Note.* All parameters are significant at *p* < .001.

All indicators had a meaningful saturation with their corresponding factor, apart from Item 5 whose saturation was somewhat low (Figure 1). A correlation of 0.40 (*p* < .001) between the two factors suggests that the MAC-RF measures two related but clearly distinct aspects of COVID fears; one describing fear of being infected with the virus, oneself or a person’s loved one, and the other describing fears related to choosing an inadequate strategy in dealing with the pandemic, including informing oneself too much or too little about the pandemic.

### Scale Reliability and Validity

In accordance with the CFA results, two subscales for MAC-RF were created. The Cronbach’s alpha for MAC-RF 1 is .77 and for the MAC-RF 2 is .65. The correlation between the two subscales is *r* = 0.29, *p* <.001.

To inspect associations with psychopathology, correlations between the MAC-RF (subscales and total score) and the CCSM (domains and total scores) were calculated. As seen in Table 3, the highest correlations were detected between the anxiety domain of the CCSM and MAC-RF, falling in the range of a moderate correlation. Other correlations were mostly small in magnitude or, in the case of suicidal ideation and psychosis, non-significant.

**Table 3:** Correlations Between the MAC-RF and CCSM (N = 346)

CCSM subscale	MAC-RF		
	1	2	Total
Depression	.23**	.30**	.33**
Anger	.24**	.27**	.31**
Mania	.01	.11*	.07
Anxiety	.27**	.40**	.40**
Somatic symptoms	.15**	.24**	.23**
Suicidal ideation	-.02	.06	.02
Psychosis	-.02	.05	.02
Sleep problems	.12*	.18**	.18**
Memory problems	.11*	.15**	.15**
Obsession/compulsion	.08	.16**	.14**
Dissociation	.09	.18**	.16**
Maladaptive personality	.08	.20**	.16**
Substance use	.09	.10	.11*
Total score	.20**	.33**	.32**

*Note.* CCSM = DSM-5 Self-Rated Level 1 Cross-Cutting Symptom Measure—Adult. MAC-RF = Multidimensional Assessment of COVID-19-Related Fears.

*p < .01. **p < .001.

To further explore the construct of fear of COVID-19, we examined the associations between the two subscales of the MAC-RF (and total score) and a similar measure of COVID-19 anxiety (CAS5), health anxiety (SHAI), cyberchondria (SCS), three aspects of anxiety sensitivity (ASI-3), depression (DASS-D), protective health behaviors (HB) and health care utilization (HCU).

As seen in Table 4, the two aspects of fear of COVID-19 measured by the MAC-RF appear to have varying associations with a number of these constructs. For example, the CAS5 captures only one aspect of fear of COVID-19 – the fear of infection – and not the second aspect - the fear of choosing the wrong strategy in coping with pandemic. This explains the fact that a correlation of only .58 was detected between the two measures. As expected, a stronger correlation was found between the MAC-RF total score and health anxiety (.39) and cyberchondria (.44) than with depression (.28). Fear of infection had a stronger correlation with health anxiety and Fear of choosing the wrong strategy in coping with pandemic had a stronger correlation with cyberchondria and depression. With respect to anxiety sensitivity, a somewhat stronger correlation was found between the social domain of ASI and the Fear of infection subscale of the MAC-RF, which includes the fear of others and for others. Furthermore, protective health behaviors were strongly correlated with Fear of infection (.64), but not with Fear of choosing the wrong strategy in dealing with pandemic (.26). Finally, neither aspect of fear of COVID-19 correlated with the number of doctor visits in the previous two months.

**Table 4:** Correlations Between MAC-RF Subscales and Related Constructs

Measure	MAC-RF			
	1	2	Total	*N*
CAS5	.65	.23	.58	477
SHAI	.33	.29	.39	346
SCS	.32	.42	.44	321
ASI physical	.28	.31	.36	346
ASI cognitive	.20	.25	.27	346
ASI social	.36	.21	.29	346
DASS-D	.17	.29	.28	346
HB	.64	.26	.58	346
HCU	.04^ns^	.05^ns^	.06^ns^	346

*Note.* MAC-RF = Multidimensional Assessment of COVID-19-Related Fears; CAS5 = COVID-19 Anxiety Scale; SHAI = Short Health Anxiety Inventory; SCS = Short Cyberchondria Scale; ASI = Anxiety Sensitivity Index; DASS-D = Depression, Anxiety and Stress Scale 21; HB = Protective Health Behaviors; HCU = Health Care Use.

All correlations are significant at *p* < .001 except correlations with HCU, which are all non-significant. With respect to the SCS, participants who reported never searching for health information online were excluded from the analyses because including these participants would obscure the definition of cyberchondria at the low end.

Finally, correlations between the MAC-RF subscales and fear of different types of consequences related to COVID-19 are presented in Table 5.

**Table 5:** Correlations Between the MAC-RF and Fear of Different Types of Consequences Related to COVID-19

Feared Consequences Related to COVID-19	*M* (*SD*)	MAC-RF		
		1	2	Total
Physical health	2.70 (1.13)	.49**	.27**	.49**
Mental health	2.74 (1.25)	.27**	.46**	.44**
Loved ones health	3.64 (1.12)	.47**	.29**	.48**
Financial loss	3.18 (1.21)	.06	.22**	.16**
Loss of civil liberties	3.10 (1.38)	-.20**	.18**	-.05
Disturbed relationships	3.07 (1.32)	.02	.28**	.16**

*Note.* MAC-RF = Multidimensional Assessment of COVID-19-Related Fears.

*p < .01. **p < .001.

The Fear of infection subscale had a stronger correlation with the fear of consequences for one’s physical health and the health of loved ones and a small negative correlation with the fear of loss of civil liberties. Conversely, Fear of choosing inadequate strategy had a stronger correlation with the fear of consequences for mental health and was also positively correlated with fear of financial loss, loss of civil liberties and disrupted relationships with others.

## Discussion

The aim of this study was to validate a theoretically based measure of COVID-19 related fear – the MAC-RF – in a Croatian sample and to further explore its validity. In contrast to the 1-factor structure reported by the authors of the scale (Schimmenti, Starcevic, et al., 2020), a 2-factor structure was revealed in the Croatian sample. With regards to the scale’s general properties, its association with general psychopathology as measured by the CCSM was similar to that reported by the authors. Furthermore, the stronger associations between the MAC-RF and health anxiety and cyberchondria than with depression found in this study further attest to its convergent and divergent validity and expands previous findings regarding the instrument. Additionally, with respect to concurrent validity, we found a moderate to strong association between the MAC-RF and a previously validated scale of COVID anxiety (MAC5).

However, total scale reliability was lower in our study (.72; original study = .84). This might be the consequence of the 2-factor structure registered in this study. Considering that each subscale has only four items, low Cronbach’s alpha (.77 and .65) is not surprising. Therefore, it would be more suitable to assess test-retest reliability. The two items from the cognitive domain showed the lowest factor saturations in both studies, suggesting that there may be issues with item formulation. Furthermore, informing oneself about COVID-19 may also be seen as an action (behavioral domain). Finally, cognitive domain is maybe too narrowly defined since knowing and not knowing can be achieved through different means besides informing oneself in an explicit way; such as through talking vs. not talking about COVID-19 or maybe even through ruminating about the information one has attained vs. suppressing it.

The two MAC-RF factors identified in this study are: Fear of infection, which reflects emotional-interpersonal feature, and Fear of choosing an inadequate strategy, which reflects cognitive-behavioral feature form the Schimmenti, Billieux, and Starcevic’s model (2020). The two factors were only moderately associated (.40), suggesting that they measure two distinct aspects of COVID-19 related fears. This is further confirmed by a somewhat different patterns of association that the two subscales shared with several relevant constructs. For example, Fear of infection has a stronger correlation with health anxiety and COVID-19 anxiety, suggesting that this factor captures negative emotional states related to COVID-19 and primarily deals with feared consequences for one’s somatic health and the health of others. This aspect of COVID-19 fear also appears to be a much stronger predictor of safety behaviors. Furthermore, this subscale is more strongly related to the social aspect of anxiety sensitivity, which may reflect the fear of embarrassment due to revealing COVID-19 anxiety.

On the other hand, Fear of choosing an inadequate strategy when dealing with pandemic has a stronger association with cyberchondria, which is itself a dysfunctional strategy for dealing with health fears. According to a recently proposed metacognitive model of cyberchondria (Fergus & Spada, 2018), the vicious cycle of excessive online health information and distress is maintained due to conflicting metacognitive beliefs about this strategy: it is deemed helpful in protecting one’s somatic health, but harmful to one’s mental health. Similarly, the second MAC-RF subscale may reflect a metacognitive aspect of COVID-19 fear (beliefs about strategies for dealing with pandemic) that is dialectical in nature: fear of doing too much or too little, reading too much or too little. Furthermore, this subscale shares the strongest correlation with the fear of consequences of pandemic for mental health. This finding, together with the dialectic nature of this subscale, may explain its weak correlation with safety behaviors. This subscale is also associated with the fear of disturbed relationships due to pandemic. Besides dealing with evaluation of one’s knowledge and action in respect with COVID-19, this subscale also deals with tolerating uncertainty so its association with this aspect needs to be explored in further studies. Finally, an important aspect of this subscale is considering responsible social action as discussed in the Schimmenti, Billieux, and Starcevic’s model (2020). Although, we did not find correlation between this subscale and safety behaviors, it seems probable that only certain items or their combination is predictive of taking action.

A lack of association between number of doctor’s visits with either of the MAC-RF subscales may be explained by the fact that some people might avoid doctors due to the fear of contracting the coronavirus, while others may go “doctor shopping” to get reassurance. Also, it should be noted that over 60% of the sample have not visited a doctor during the last two months.

The different underlying structure of the MAC-RF in our sample may suggest cultural, social or economic differences, but might also be due to fact that the two studies were conducted in different epidemiological circumstances. In the original study (Schimmenti, Starcevic, et al., 2020), data was collected a month after restrictions were lifted, while our data was collected during a period in which new restrictions were being imposed and a significant growth in new cases was being registered. It is possible that there are differences in the definition of this construct depending on epidemiological circumstances. Further studies should examine a bifactor structure for the MAC-RF having in mind that both a single and two-factor structure may co-exist and may both have a meaningful interpretation as suggested for other psychopathology constructs (Bornovalova et al., 2020).

Finally, the different instruments developed in this field capture different aspects of problematic psychological reactions to COVID-19. While some emphasize emotional (Lauri-Korajlija & Jokić-Begić, 2020), behavioral (Nikčević & Spada, 2020) or physiological (Lee, 2020) aspects, others encompass a combination of emotional, physiological, cognitive and behavioral components (e.g., Taylor et al., 2020; Ahorsu et al., 2020). Others even go beyond the fear of illness and include fear of economic consequences (Arpaci et al., 2020). An instrument’s scope will certainly affect its associations with other relevant constructs: predictors, mediators and outcomes of fear. Developing a theoretical approach to COVID-19 related distress can help in achieving a consistent definition of this construct (or constructs), developing adequate measurement tools, integrating knowledge form different studies, and developing targeted interventions.

This study supports the dialectical nature of COVID-19 fears (Schimmenti, Billieux, & Starcevic, 2020), since items describing opposing fears reflect a single construct, and attests to the complexity of human experiences in the time of a global health crisis. Several strategies for addressing COVID-19 anxiety have been suggested by the authors of MAC-RF (Schimmenti, Billieux, & Starcevic, 2020); practicing mindfulness to improve appraisal of the body and to adopt acceptance and self-compassion, delivering targeted interventions to foster attachment security, using strategies to improve emotion regulation, and promoting responsibility. The results of this study further emphasize that treatment might need to focus not only on fear of becoming infected, but also on a metacognitive aspect that reflects conflicting beliefs about strategies used when dealing with the pandemic. Using a combination of cognitive continuum and listing advantaging and disadvantages of extreme strategies (e.g., reading about COVID too much or not at all), as a form of cognitive restructuring within cognitive-behavioral therapy, could help adopting appropriate intensity of health-related behaviors. It may also be necessary to modify metacognitive beliefs about strategies in dealing with pandemic and practice tolerating uncertainty which fuels COVID-19 anxiety.

The disadvantages of this study that place limits on its findings include: a non-representative sample (certain groups are underrepresented), a self-selection bias (people more affected by COVID-19 might have been more likely to participate) and the cross-sectional design (no causal associations can be claimed).

### Conclusions

This study suggests that the MAC-RF might be a useful instrument in assessing COVID-19 fears. In a Croatian sample and at a time of a rapid increase in daily cases, this instrument appears to measure two distinct, but related factors: Fear of infection (emotional aspect) and Fear of choosing an inadequate strategy when dealing with pandemic (metacognitive aspect). Further studies using the MAC-RF across different cultures and different epidemiological circumstances are needed.

## Supplementary Materials

The research data is collected in the validation study of Multidimensional Assessment of COVID-19-Related Fears (MAC-RF) – Croatian version. A total of 477 participants completed the online survey during the COVID-19 pandemic. For access see Index of Supplementary Materials below.

10.23668/psycharchives.5408 Supplement 1 Supplementary materials to "Fear of becoming infected and fear of doing the wrong thing – Cross-cultural adaptation and further validation of the multidimensional assessment of COVID-19-related fears" [Research data]

BagarićB.
Jokić-BegićN.
 (2022). Supplementary materials to "Fear of becoming infected and fear of doing the wrong thing – Cross-cultural adaptation and further validation of the multidimensional assessment of COVID-19-related fears"
[Research data]. PsychOpen. 10.23668/psycharchives.5408
PMC966734336397743

## Data Availability

For this article, a data set is freely available (Bagarić & Jokić-Begić, 2022)
